# The Vietnamese version of the nursing critical thinking in clinical practice questionnaire: Translation and psychometric evaluation

**DOI:** 10.1002/nop2.834

**Published:** 2021-03-11

**Authors:** Tuan Van Nguyen, Hsueh‐Erh Liu

**Affiliations:** ^1^ Faculty of Nursing and Medical Technology Can Tho University of Medicine and Pharmacy Can Tho Vietnam; ^2^ School of Nursing College of Medicine Chang Gung University Taoyuan Taiwan, ROC; ^3^ Department of Rheumatology Chang Gung Memorial Hospital Taoyuan Taiwan, ROC; ^4^ Department of Nursing College of Nursing Chang Gung University of Science and Technology Taoyuan Taiwan, ROC

**Keywords:** critical thinking, N‐CT‐4 Practice, nursing, psychometric properties, Vietnamese

## Abstract

**Aims:**

This study translated and evaluated the validity and reliability of the Vietnamese version of the Nursing Critical Thinking in Clinical Practice Questionnaire (N‐CT‐4 Practice (V‐v)).

**Design:**

Forward‐ and back‐translation approach developed by Sousa and Rojjanasrirat (2011).

**Methods:**

545 nurses were recruited based on convenience sampling and asked to complete the N‐CT‐4 Practice (V‐v) questionnaire for psychometric testing. Data were collected during June 2019 in three public hospitals located in Southwestern Vietnam. We evaluated translation equivalence, the item content validity index, floor/ceiling effects, construct validity, internal consistency reliability and test–retest reliability.

**Results:**

The N‐CT‐4 Practice (V‐v) questionnaire retained the meaning of the original English version and was clear, explicit and easy for nurses to understand. The item content validity index was 1.0. There were no floor/ceiling effects. The Cronbach's alpha was 0.98. The intraclass correlation coefficient was 0.81. Confirmatory factor analysis indicated that this Vietnamese version fit the proposed model.

## 1 INTRODUCTION

The variety of populations around the world indicates a critical need for cross‐culturally validated studies questionnaires or scales. Healthcare researchers and clinicians must have access to reliable and valid instruments of an interesting concept in their own cultures and languages to perform cross‐cultural studies and/or deliver the quality of patient care (Sousa & Rojjanasrirat, 2011). Moreover, the development of a questionnaire requires spending of time and money. First, creating the questionnaire and selecting domains and items that will best investigate the construct of interest. Second, validating and ensuring of the questionnaire measures what it is purposive to measure (Epstein et al., 2015). Therefore, using previously developed questionnaires with good psychometric properties can save time and endeavour. However, these questionnaires need to be valid as culturally accepted and appropriately translated (Cha et al., 2007). Consequently, the process of translation and psychometric evaluation becomes an essential part of cross‐cultural studies.

## 2 BACKGROUND

Critical thinking has been described from multidisciplinary perspectives, with disparate definitions related to the concepts of cognition, attitude, process and skills. Many nursing scholars agree that critical thinking is necessary and valuable in nursing education and practice, even though there is no clear consensus on a definition (Chan, 2013; Mundy & Denham, 2008; Shoulders et al., 2014; Zuriguel‐Pérez et al., 2015). In nursing practice, critical thinking is a cognitive process that represents the competence to apply reasoning with the desire to decrease errors in decision‐making (Alfaro‐Lefevre, 2016; Chao et al., 2013; Shinnick & Woo, 2013). Many professional organizations recognize and support that critical thinking is an essential element in the role of nursing (Brunt, 2005; Mundy & Denham, 2008; Simpson & Courtney, 2002). Nurses apply critical thinking skills daily to assess, plan and provide quality patient care (Bambini et al., 2009). Besides, critical thinking ability has been associated with clinical decision‐making (Bowles, 2000; Brooks & Shepherd, 1990; Lee et al., 2017; Ludin, 2018; Martin, 2002), nursing competence (Chang et al., 2011), nursing processes (Bittencourt & Crossetti, 2013; Chabeli, 2007; Huckabay, 2009), nurse workplace production and problem‐solving ability (Lunney, 2010), and research utilization (Profetto‐McGrath et al., 2003).

The measurement of the level of critical thinking ability has been a concern of numerous studies in the last several decades (Zuriguel‐Pérez et al., 2017). Based on a review of 34 studies, sixteen different tools were identified for measuring the concept of critical thinking. The most commonly used instruments are standardized, such as the California Critical Thinking Disposition Inventory (CCTDI), California Critical Thinking Skills Test (CCTST) (Facione & Facione, 1992), Health Sciences Reasoning Test (HSRT) (Facione et al., 2010) and Watson‐Glaser Critical Thinking Appraisal (WGCTA) (Watson, 1980). However, the review found that most of the instruments were used in nursing education but not in nursing clinical practice (Carter et al., 2015). Moreover, Carter et al. (2015) also found that there was limited reporting of the reliability and validity of tools and inconsistent findings across several studies, leading to doubt about the validity of these tools in nursing contexts.

The Nursing Critical Thinking in Clinical Practice (N‐CT‐4 practice) questionnaire is a relatively newly developed self‐administered questionnaire that was designed to measure the critical thinking ability of nurses who work in clinical areas. The psychometric testing of the original N‐CT‐4 Practice questionnaire showed good validity and reliability (Zuriguel‐Pérez et al., 2017). This instrument has been translated into Turkish (URHAN & Seren, 2019) and Persian (FallahNezhad & Ziaeirad, 2018). However, the instrument was not available in Vietnam, which has extensive nursing work in the clinical area. Therefore, this study aimed to translate the English N‐CT‐4 Practice Questionnaire into Vietnamese (N‐CT‐4 Practice (V‐v)) and to examine its validity and reliability with a sample of Vietnamese clinical nurses.

## 3 METHODS

### 3.1 Translation process

The forward‐ and back‐translation process was adopted from principles established in previously published guidelines (Sousa & Rojjanasrirat, 2011). The process included 5 steps: (a) the survey was forward‐translated into Vietnamese by two independent bilingual translators; (b) a committee approach among the two translators and the bilingual researcher was used to obtain consensus on the final Vietnamese version; (c) the Vietnamese version was blindly back‐translated into English by two different independent bilingual translators, and again, the committee approach and consensus were reached on the English back‐translation version; (d) the English back‐translation version was compared with the original English version by two independent native English‐speaking experts. These experts evaluated whether the meaning of these two versions was similar using a five‐point Likert scale *(1 = strongly disagree, 2 = disagree, 3 = neutral, 4 = agree and 5 = strongly agree)*. If uncertainties and differences could not be resolved, steps 1 through 4 were repeated. (e) The Vietnamese version was sent to three experts who were familiar with the clinical nursing setting. They were asked to judge each item of the instrument for translation and content equivalence using a 4‐point Likert scale *(1 = not relevant, 2 = unable to assess relevance; 3 = relevant but needs minor alteration; and 4 = very relevant and succinct)*. The proportion of the experts’ agreement was used for an equivalent assessment of the translated instrument.

### 3.2 Psychometric evaluation

The validity of the N‐CT‐4 Practice (V‐v) was assessed with both contents and construct validity. The method suggested by Lynn (1986) and Polit et al. (2007) was used to identify the content validity on the item level content validity index (I‐CVI) (Lynn, 1986; Polit et al., 2007). Three experts assessed the relevance of each item on a 4‐point Likert scale, from (1) not relevant to (4) very relevant. Lynn (1986) stated that the I‐CVI must be 1.0 when there were five or fewer experts.

Floor and ceiling effects are computed by the percentage frequency of the lowest or highest possible score gained by participants. The 15% threshold was used to determine the percentage of the sample that has the lowest and the highest scores of the N‐CT‐4 Practice (V‐v), and its subscales were adopted to clarify the ceiling and floor effects (Terwee et al., 2007).

The construct validity was evaluated by confirmatory factor analysis (CFA). The CFA was performed with structural equation modelling, and the estimation of parameters was done using the maximum likelihood model. Model fit was explored with several procedures because different authors have recommended using several indicators to identify the fit of models (Hu & Bentler, 1999; Schreiber et al., 2006).

The reliability of N‐CT‐4 Practice (V‐v) was evaluated by both internal consistency and test–retest reliability. The former was assessed with Cronbach's alpha coefficient. The scale was considered to display acceptable, good, or excellent internal consistency when this index was more than 0.7, 0.8 or 0.9, respectively. The latter was evaluated by the intraclass correlation coefficient (ICC), and a minimum value of 0.7 was considered satisfactory (Terwee et al., 2007).

### 3.3 Setting, participants and procedure for data collection

Data collection was performed during June 2019 in three representative public hospitals located in the southwestern part of Vietnam, which are as follows: Can Tho Central General Hospital; Can Tho General Hospital; and Can Tho University of Medicine and Pharmacy Hospital. They provide similar healthcare quality to people around that area and have an essential connection in the national Vietnam medical network. The participants were clinical nurses recruited from the internal medicine, surgery, intensive care unit (ICU), emergency department (ED), and anaesthesiology and recovery department in these hospitals. Regarding estimate the sample size in factor analysis, the 5–10 participants per variable guideline are commonly suggested (Floyd & Widaman, 1995), and a number of 100 is “poor,” 200 is “fair,” 300 is “good,” 500 is “very good,” and 1,000 or more is “excellent” (Matsunaga, 2010). In this study, the required sample size was 545, with 5 participants per variable, which was treated as one item in the N‐CT‐4 Practice (V‐v) questionnaire (109‐item). Convenience sampling was conducted to recruit the sample. The eligibility criteria for nurses included (a) work as a clinical nurse; (b) 18 years old and above; and (c) full‐time employment. Participants who were absent during data collection, such as sick leave or maternity leave, were excluded.

The researcher contacted three hospitals and obtained a name list of nurses from each hospital. The research group contacted and invited these nurses to participate in this study. The research participants were provided both verbal and written information relating to the purpose, benefits, and risks of research as well as procedures to assure anonymity, confidentiality, and voluntary participation to potential subjects. Once they agreed, the consent form was signed, and a questionnaire was sent to them directly. It took approximately 20 min for participants to complete the N‐CT‐4 Practice (V‐v) questionnaire and provide demographic characteristics.

### 3.4 Instruments

The N‐CT‐4 Practice questionnaire was developed by Zuriguel‐Pérez (2017) and based on the theoretical model of Alfaro‐LeFevre (2016). It was a specific tool developed to measure the level of critical thinking ability of nurses in clinical practice environments. This scale has 109 items with a 4‐point Likert response format (1 = never or almost never, 2 = occasionally, 3 = often and 4 = always or almost always). There are four dimensions: personal characteristics (Prs, 39 items); intellectual and cognitive abilities (Int, 44 items); interpersonal abilities and self‐management (Atg, 20 items); and technical abilities (Tcn, 6 items). The total score ranges between 109–436, and the levels of critical thinking are categorized as low level (score <329), moderate level (score between 329–395) and high level (score >395). By expert evaluation, the original results from 399 clinical nurses had an I‐CVI of 0.85, a total Cronbach's alpha coefficient of 0.96 and an ICC of 0.77. The goodness‐of‐fit indices in CFA were *χ* ^2^/*df* = 1.95, RMSEA = 0.055, SRMR = 0.65, CFI = 0.629 and TLI = 0.621, indicating that the N‐CT‐4 Practice was in keeping with the four‐dimensional model proposed by Alfaro‐Levre (Zuriguel‐Pérez et al., 2017).

### 3.5 Statistical analysis

The Statistical Package for the Social Sciences (SPSS) for Window version 22.0 (IBM Corp.) was used to analyse the data. Descriptive statistics were used to summarize the characteristics of the participants. The I‐CVI was calculated to assess the content validity of the N‐CT‐4 Practice (V‐v) using Microsoft Excel. CFA was conducted using the Analysis of Moment Structures (AMOS) version 22.0 to evaluate the construct validity. The goodness‐of‐fit of the model was assessed by using the indices and criteria: chi‐square test (*χ* ^2^; non‐significant). Because chi‐square is sensitive to sample size, we evaluated the goodness‐of‐fit index based on the ratio between chi‐square and the degrees of freedom (*χ* ^2^/*df*; <3), the root mean square error of approximation (RMSEA; <0.06), the standardized root mean square residual (SRMR; <0.08), the comparative fit index (CFI >0.95 is a good fit) and the Tucker–Lewis index (TLI > 0.95 is a good fit; 0 < TLI < 1 can be acceptance) (Hu & Bentler, 1999; Schreiber et al., 2006). The Cronbach's alpha coefficient was used to evaluate the internal consistency, and a value of *α* ≥ 0.7 was acceptable. The ICC (two‐way mixed effects model) was used to assess the test–retest reliability for 2 weeks, and the value of ICC ≥ 0.7 was satisfactory (Terwee et al., 2007).

### 3.6 Ethical considerations

This study adhered to the ethical principles in congruence with the Declaration of Helsinki adhered (Helsinki Declaration, 2013) and was permitted ethical approval by the ethical review board of the first author's institution (No: 1658/QĐ‐ĐHYDCT).

## 4 RESULTS

### 4.1 Characteristics of participants

The questionnaire was completed by 545 clinical nurses. Overall, the majority were female (71.4%), and half of nurses were married (50.5%). The age and years of work experience ranged from 21–60 years (median = 29) and 1–41 years (median = 5), respectively. Most of the nurses had diplomas and associate degrees (73.9%), followed by bachelor's and graduate degrees (26.1%). The present working areas of the samples were internal medicine (38.5%), surgery (33.4%) and critical care units (28.1%).

### 4.2 Translation equivalence

All three bilingual experts conducted rating independently, and the results showed that the N‐CT‐4 Practice (V‐v) questionnaire retained the meaning of the original English version and that the language used in the Vietnamese version was clear, explicit and easy for nurses to understand. The original English version and the back‐translated English version were compared by two native English speakers. They strongly agreed and agreed that the back‐translated version preserved the equivalent meaning of the original English version for all items (I‐CVI = 1.0). Only one item of the back‐translated English version needed to be modified. Specifically, the English version was “I recognize my own emotions,” and the back‐translation was “I recognize my emotion.” Emotions are plural, meaning more than one emotion. These experts suggested modifying it. However, nouns in Vietnamese do not distinguish singular or plural, so the two sentences mentioned above have the same meaning in Vietnamese language.

### 4.3 Reliability

The overall Cronbach's alpha coefficient of the N‐CT‐4 Practice (V‐v) was 0.98, showing excellent internal consistency (Terwee et al., 2007; Waltz et al., 2017). The Cronbach's alpha for the four subscales ranged from 0.86–0.97, indicating the good reliability of each subscale. The ICC for the N‐CT‐4 Practice (V‐v) was 0.81, and the ICC for the four subscales ranged from 0.76–0.86 (*p* < .001), indicating good stability for the 2 weeks (Table 1).

**TABLE 1:** Reliability and construct validity of the Vietnamese and original English versions

Value	N‐CT−4 practice (V‐v)						N‐CT−4 practice			
	Floor/ceiling effect		Cronbach's *α*	ICC	CFA		Cronbach's *α*	ICC	CFA	
	FE, *N* (%)	CE, *N* (%)								
Total score	1 (0.2)	11 (2.0)	0.98	0.81	*χ* ^2^/*df*	2.87	0.96	0.77	*χ* ^2^/*df*	1.95
Subscale: Prs	1 (0.2)	12 (2.2)	0.95	0.86	RMSEA	0.059	0.89	0.70	RMSEA	0.055
Int	1 (0.2)	34 (6.2)	0.97	0.76	SRMR	0.063	0.94	0.77	SRMR	0.65
Atg	1 (0.2)	51 (9.4)	0.95	0.80	CFI	0.73	0.86	0.84	CFI	0.629
Tcn	1 (0.2)	70 (12.8)	0.86	0.84	TLI	0.72	0.78	0.76	TLI	0.621

### 4.4 Content validity

The N‐CT‐4 Practice (V‐v) questionnaire had an excellent item level content validity index (I‐CVI = 1.0), indicating that all items were scored as acceptable. However, the three experts suggested that the N‐CT‐4 Practice (V‐v) should be shorter to help nurses focus their attention and maintain concentration while answering the 109 items.

### 4.5 Floor and ceiling effects

There were no floor and ceiling effects (<15%) for the total score and four subscales of the N‐CT‐4 Practice (V‐v) (Table 1).

### 4.6 Construct validity

**FIGURE 1:**
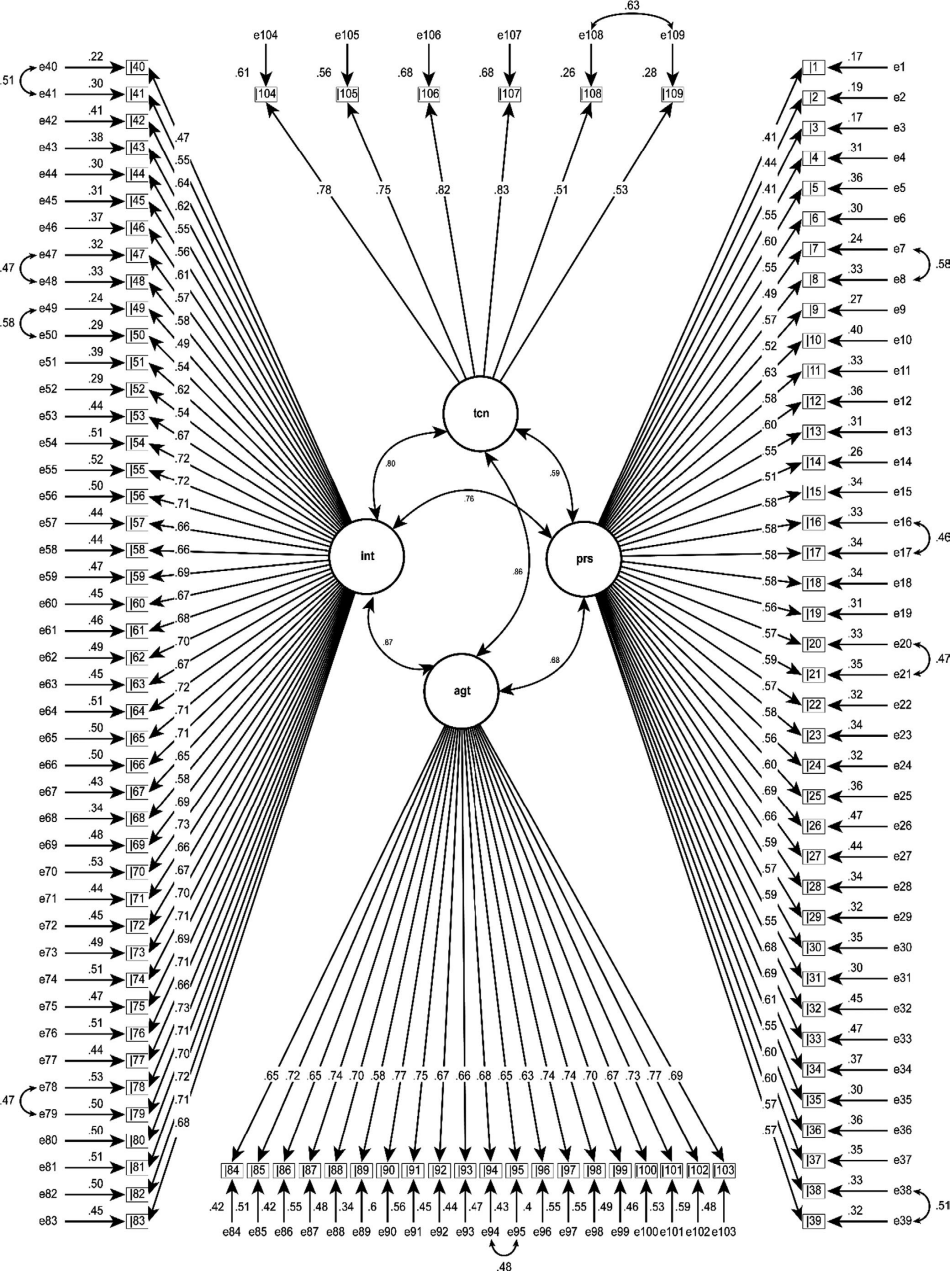
Confirmatory factor analysis model for the N‐CT‐4 practice (V‐v)

All the values for estimated parameters for the model were statistically significant in all cases (*p* < .001), consistent with what was expected. None of the variances or correlations revealed values considered to be unsuitable to the extent that the proposal would be invalidated. However, the basic model was not satisfied with *χ* ^2^/*df* = 3.17, RMSEA = 0.063, CFI = 0.69 and TLI = 0.68. Therefore, the modification indices recommended that the fit would be better when the residuals between items 7 and 8, 16 and 17, 20 and 21, 38 and 39, 40 and 41, 47 and 48, 49 and 50, 78 and 79, 94 and 95, and 108 and 109 were correlated. The graphic representation (path diagram) of the model is shown in Figure 1. Convention dictates that squares declare measured variables (e.g. i1 is item 1 in the N‐CT‐4 Practice (V‐v) questionnaire and that circles indicate latent variables (e.g. personal dimension, intellectual dimension). The value that is revealed with the single‐headed arrows between the circles and the squares shows the factor loading; the double‐headed arrows show the correlations between pairs of variables. CFA reported that the correlation between the pairs of variables in the model—personal and intellectual, personal and interpersonal, personal and technical, intellectual and interpersonal, intellectual and technical, and interpersonal and technical—was 0.76, 0.68, 0.59, 0.87, 0.80, 0.86, respectively (*p* < .001), indicating that four dimensions of the model are adequate.

The results of the chi‐square test showed that the assumption of a perfect model needed to be rejected (*χ* ^2^ = 16,569.06; *p* < .0001), indicating that the fit of the data to the hypothesis of a perfect model was not entirely satisfied. The adjusted indices based on covariance showed optimal values: *χ* ^2^/*df* = 2.87, RMSEA = 0.059, SRMR = 0.063, although the incremental measurement indices produced values below the level of good model fit: CFI = 0.73, and TLI = 0.72 (Table 1). Overall, the findings of the goodness‐of‐fit indices indicated that the structure of the proposed questionnaire is acceptable.

## 5 DISCUSSION

The N‐CT‐4 Practice questionnaire is a new scale used to measure the level of critical thinking ability of nurses in their daily practice, and both the Spanish and English versions show good psychometric properties (Zuriguel‐Pérez et al., 2017). This is the first translation to obtain the Vietnamese version of the N‐CT‐4 Practice and verify its psychometric properties. The results showed that the N‐CT‐4 Practice (V‐v) questionnaire has satisfactory psychometric properties. Overall, the N‐CT‐4 Practice (V‐v) had good translation equivalence, good and excellent internal consistency, no floor and ceiling effects, and excellent I‐CVI. It has similar goodness‐of‐fit indices in CFA values to the original English version.

The process of forward‐ and back‐translation was performed fluently in this study, and we only slightly modified the content of some items (items 15, 31, 81 and 83). Particularly, item 15 changed the phrases of “khó khăn để vượt qua” to “thách thức để vượt qua”; item 31 changed the words “càng lớn” to “càng nhiều”; item 81 changed the phrases “làm thế nào để tôi trau dồi nó” to “cách thức tôi đã tìm hiểu”; and item 83 changed the words “cơ quan” to “tổ chức”. The findings of the I‐CVI suggested that all items were scored as satisfactory. Besides, none of the floor and ceiling effects indicated that the N‐CT‐4 Practice (V‐v) had good content validity. Consequently, nurses with the lowest or highest possible score can be distinguished from each other, thus reliability is increased (Terwee et al., 2007).

Regarding the reliability of the questionnaire, the total Cronbach's alpha coefficient had excellent qualifies (*α* = 0.98), which was similar to the values in the original English version (*α* = 0.96) (Zuriguel‐Pérez et al., 2017) and Turkish version (*α* = 0.98) (URHAN & Seren, 2019). All four subscales also had good and excellent internal consistency (ranging from 0.86–0.97), which was consistent with the original English version (ranging from 0.78–0.94) (Zuriguel Pérez, 2016) and Turkish version (ranging from 0.82–0.96) (URHAN & Seren, 2019). Moreover, the findings of test–retest reliability for the overall scale (ICC = 0.81) and the four subscales (ranging between 0.76–0.86) indicated that the N‐CT‐4 Practice (V‐v) possesses good stability over time, which was consistent with the original English version (Zuriguel Pérez, 2016; Zuriguel‐Pérez et al., 2017).

In this study, the results from a CFA on data from 545 clinical nurses indicated that the psychometric properties of the N‐CT‐4 Practice (V‐v) questionnaire are satisfactory. Specifically, most of the values used to evaluate the goodness‐of‐fit are satisfactory (*χ* ^2^/*df* = 2.87, RMSEA = 0.059, SRMR = 0.063 and TLI = 0.72). However, the value of CFI was only close to the appropriate level (Schreiber et al., 2006). These findings are consistent and somewhat better than those for the original English version, which reported a very high SRMR value (SRMR = 0.65 > 0.08) (Table 1), and similar with Turkish version (*χ* ^2^/*df* = 2.07, RMSEA = 0.063, SRMR = 0.065, CFI = 0.63 and TLI = 0.63) (URHAN & Seren, 2019). These findings also confirmed that the N‐CT‐4 Practice (V‐v) questionnaire is consistent with the 4‐Circle Critical Thinking Model of Alrafo‐LeFevre (2016), which was the theoretical basis of the N‐CT‐4 Practice questionnaire. Therefore, the psychometric properties of the N‐CT‐4 Practice (V‐v) questionnaire are satisfactory and can be applied to examine the level of critical thinking ability in Vietnamese clinical nurses.

A limitation of this study was that the samples were recruited from three public hospitals in the southwestern part of Vietnam and may not fully represent all nurses in Vietnam. However, the large sample size did represent the availability of this tool in Vietnam in general.

## 6 CONCLUSIONS

In summary, the current study provides evidence that the N‐CT‐4 Practice (V‐v) questionnaire has acceptable reliability and validity and can be used to assess the level of critical thinking ability in Vietnamese clinical nurses. Therefore, nurse managers and educators can apply this scale to assess the level of critical thinking ability of clinical nurses in the future.

The use of the N‐CT‐4 Practice (V‐v) questionnaire supplies a valuable opportunity to evaluate critical thinking ability in nursing practice and produce additional opportunities for cross‐cultural comparison studies between Vietnamese and other countries. Thus, further exploration and training associated with critical thinking can be achieved by using this valid instrument.

## CONFLICT OF INTEREST

The authors have no conflicts of interest to report.

## Data Availability

The data that support the findings of this study are available from the corresponding author upon reasonable request.
